# Modeling the transmission dynamics of Ebola virus disease in Liberia

**DOI:** 10.1038/srep13857

**Published:** 2015-09-08

**Authors:** Zhi-Qiang Xia, Shi-Fu Wang, Shen-Long Li, Liu-Yu Huang, Wen-Yi Zhang, Gui-Quan Sun, Zhong-Tao Gai, Zhen Jin

**Affiliations:** 1 Complex Systems Research Center, Shanxi University, Taiyuan, Shanxi 030006, P. R. China; 2 Department of Mathematics, North University of China, Taiyuan, Shanxi 030051, P. R. China; 3 Department of Children’s Medical Laboratory Diagnosis Center, Qilu Children’s Hospital of Shandong University, Jinan 250022, P. R. China; 4 Institute of Disease Control and Prevention, Academy of Military Medical Science, Beijing, 100071, P. R. China; 5 Department of Pediatrics, Qilu Children’s Hospital of Shandong University, Jinan 250022, P. R. China

## Abstract

Ebola virus disease (EVD) has erupted many times in some zones since it was first found in 1976. The 2014 EVD outbreak in West Africa is the largest ever, which has caused a large number of deaths and the most serious country is Liberia during the outbreak period. Based on the data released by World Health Organization and the actual transmission situations, we investigate the impact of different transmission routes on the EVD outbreak in Liberia and estimate the basic reproduction number *R*_0_ = 2.012 in the absence of effective control measures. Through sensitivity and uncertainty analysis, we reveal that the transmission coefficients of suspected and probable cases have stronger correlations on the basic reproduction number. Furthermore, we study the influence of control measures (isolation and safe burial measures) on EVD outbreak. It is found that if combined control measures are taken, the basic reproduction number will be less than one and thus EVD in Liberia may be well contained. The obtained results may provide new guidance to prevent and control the spread of disease.

Ebola is a highly pathogenic virus, and the mortality of EVD is about 50–90%^1^. Patients who infected by Ebola virus may have the symptoms of headaches, vomiting, diarrhoea and so on^2^,^3^,^4^,^5^. It was first found in 1976^2^,^6^ and has attracted lots of researchers to focus on it. However, its natural reservoirs have not been well identified until now^2^,^6^,^7^,^8^. The main route of infection for EVD is direct contact with the patients’ bodily fluids, including blood, sweat, vomit, excrement, urine, saliva, or semen and so on^2^,^3^,^4^,^6^,^9^. The incubation period of EVD is about 2 ~ 21 days and the patients in the incubation period are not infectious^10^,^11^,^12^,^13^,^14^,^15^.

The outbreak of EVD in 2014 started from Guinea, then spread to West Africa^11^, of which the most serious region is Liberia. Until November 14, 2014, the World Health Organization had reported 14,415 cases, and 5,506 cases died. Based on the actual situations, it was found that absence of effective control measures was the main cause for Ebola outbreak. Moreover, severe shortage of medical resources^16^,^17^ and traditional funerals^18^ may result in the persistence of EVD. In other words, effective measures for EVD control are still lack, which needs to be paid more attention by medical staffs, epidemiologists, mathematicians and so on.

Mathematical modeling is one of the most important tools in analyzing the epidemiological characteristics of infectious diseases and can provide some useful suggestions. Various models have been used to study different aspects of EVD. Chowella *et al.* constructed a dynamical model for EVD transmission (Congo 1995 and Uganda 2000) and fitted it to historical data in estimation of *R*_0_^19^. Althaus presented a susceptible-exposed-infectious-recovered (SEIR) model and fitted the model to the reported data of infected cases and deaths for EVD in Guinea, Sierra Leone and Liberia^20^. Legrand *et al.* considered different settings for transmission (in the community, in the hospital, during burial ceremonies) for EVD in the estimation of *R*_0_^10^. Camacho *et al.* divided the incubation population into two categories for EVD^21^. Lewnard *et al.* investigated the impact of the EVD with limited medical resources^16^. However, in the real situations, the infective case of Ebola should be divided into two classes: suspected case (*I*_*S*_) and probable case (*I*_*P*_)^22^, which has been generally overlooked despite its potential epidemiological reality and intrinsic theoretical interest. Consequently, we propose a more actual compartmental model to describe the transmission dynamics of EVD in Liberia.

In order to understand the transmission mechanism of EVD in Liberia and search for effective control measures, we build a mathematical model to study the spread of EVD among human beings. Based on the fitting method, we perform the parameters estimation and obtain the basic reproduction number in the absence of effective control measures. What is more, we analyze the peak arrival time of disease and correlation between the related parameters and basic reproduction number *R*_0_. Additionally, we compare the efficiency of different control measures, including isolation and safe burial.

## Results

In epidemiological research, there exists a threshold parameter: basic reproduction number *R*_0_, which is denoted as the average number of secondary infections caused by a single infected agent, during his/her entire infectious period, in a completely susceptible population^23^.

### Estimation of Basic Reproduction Number *R*_0_ of EVD in Liberia

**Figure 1:**
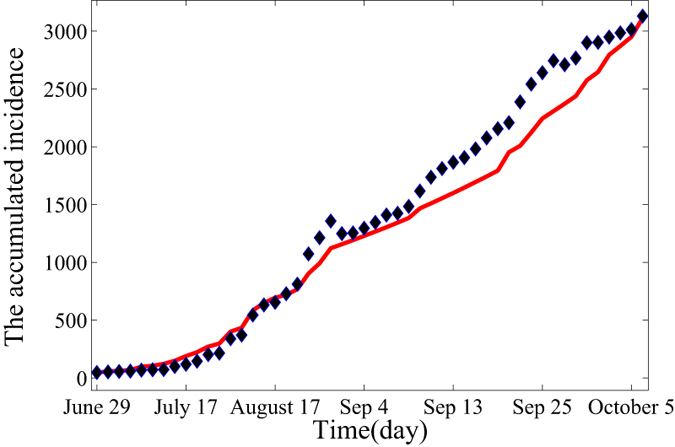
Fitting results for real data of EVD from June 29, 2014 to October 7, 2014 with the deterministic model (3), where blue asterisks are real data obtained from^24^. Estimated basis reproduction number is 2.012, which is consistent with the real cases in Liberia. This figure indicates that EVD will spread as an endemic in the absence of the control measures.

In this part, we use the least-square fitting method to estimate parameter values in order to minimize the sum of squared errors between the actual data and solution of the equation (1). The accumulated number of infected cases with time *N*_*I*_(*t*) can be given by the following equation with *N*_*I*_(*t*) = *I*_*P*(c)_(*t*) + *I*_*S*(c)_(*t*), where *I*_*P*(c)_(*t*) and *I*_*S*(c)_(*t*) denote the cumulants of the *I*_*P*_ (suspect cases) and *I*_*S*_ (probable cases), respectively:
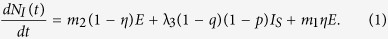


The actual number of the 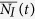 (the accumulated incidence) can be found in^24^. The estimation process is as follows: we construct a function 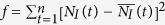 and find the suitable parameters value to make *f* to be least, where *n* is the number of actual data (In our model, *n* = 50). Biological meanings of parameters can be found in Table 1. By applying the real data in^24^ and the equation (1), we can estimate the value of *p* = 0.1, *β*_1_ = 0.1102, *β*_2_ = 0.12 and *r*_*i*_ = 0.0667. The fitting result for the accumulated incidence is given in Fig. 1.

The time range is from June 29, 2014 to October 7, 2014. As seen from Fig. 1, we can find that the slope of the fitting curve gradually increases which implies that epidemic is still aggravating. If control measures are not taken effectively, outbreak of EVD in Liberia is inevitable.

Based on the estimated values of parameters and the expression of *R*_0_:
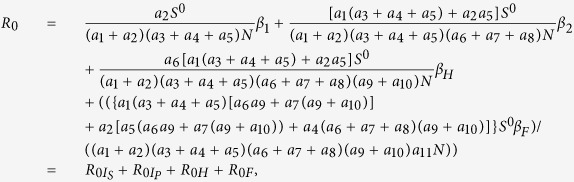
we estimate the *R*_0_ = 2.012 for the outbreak of EVD in Liberia in 2014. To be more precise, we have that 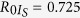, 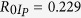, *R*_0*H*_ = 0.294 and *R*_0*F*_ = 0.736.

### Sensitivity and Uncertainty Analysis of Basic Reproduction Number *R*_0_

It is well known that the basic reproduction number (*R*_0_) determines whether the epidemic will persist or not. If *R*_0_ > 1, the disease will be epidemic; otherwise, it will eventually vanish. As a result, it is meaningful to discuss the sensitivity and uncertainty analysis of *R*_0_. In our model, several crucial parameters (*β*_1_, *β*_2_, *β*_*H*_, *β*_*F*_, *θ* and *η*) determine the value of *R*_0_. For the sensitivity and uncertainty analysis, we adopt Latin hypercube sampling (LHS) to study the influence of parameters on *R*_0_. We randomly choose 1000 samples and the six parameters follow a normal distribution.

On the basis of the 1000 samples, we can perform an analysis of *R*_0_ by computing variable partial rank correlation coefficient (PRCC). Larger the absolute value of PRCC, denotes stronger correlation between the chosen parameters and *R*_0_. The values of PRCC are showed in Table 2, and it is obvious that the absolute value of PRCC for parameter *β*_2_ is the largest, which indicates that *β*_2_ is the most influential in determining the value of *R*_0_. Although the values of *β*_*H*_, *β*_*F*_, *θ*, *η* are less important for PRCC in contrast with *β*_2_, these parameters also have some impacts on the value of *R*_0_. Furthermore, we can find that the value of PRCC between *β*_1_ or *β*_2_ and *R*_0_ is larger than those in *β*_*H*_ or *β*_*F*_, indicating that contact transmission with infected cases (*I*_*S*_, *I*_*P*_) in the community posses larger influence than contact transmission with the hospitalized cases or cases dead but not yet buried.

Additionally, we find that there are positive correlations between *β* (*β*_1_, *β*_2_, *β*_*H*_, *β*_*F*_) and *R*_0_, which suggests that the bigger the transmission coefficients, the larger value of *R*_0_. The negative correction between *θ* and *R*_0_ indicates that increasing the patients’ hospitalization rate is an effective method for controlling EVD in Liberia under the current situation.

### Peak arrival time of EVD in Liberia

In this part, we show the relationship between the parameters (typically, we just show the results of parameters *β*_1_ and *β*_2_) and the peak arrival time of the EVD as well as the maximum value of *I*_*P*_(*t*).

**Figure 2:**
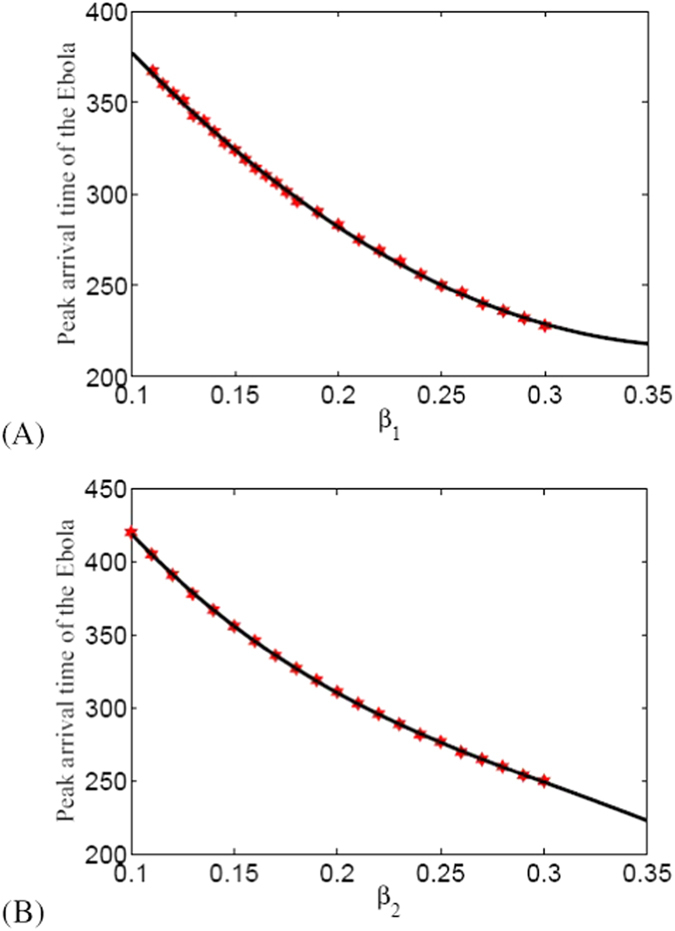
Peak arrival time of EVD with respect to *β*_1_ and *β*_2_. In our results, we estimated that EVD in Liberia may arrival its peak value after 370 days from the day when the first infected case was detected.

In Fig. 2, it is obvious that *β*_1_ (or *β*_2_) and the peak arrival time of EVD is close to linear increment relationship. This figure illustrates that the peak arrival time arrives sooner when the *β*_1_ (or *β*_2_) is larger. In other words, we can take measures to decrease *β*_1_ (or *β*_2_) to cause the delay of peak arrival time and thus less people will be infected by EVD.

**Figure 3:**
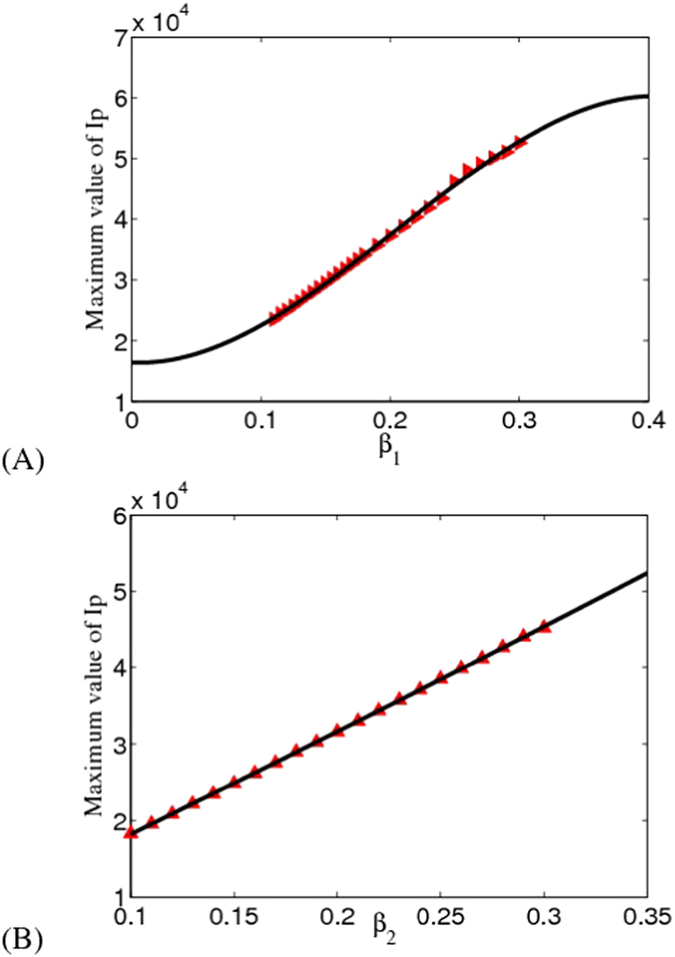
Final size of *I*_*p*_ with respect to *β*_1_ and *β*_2_. The figure suggests that probable infectives of EVD in Liberia may reach 22, 000 cases.

In Fig. 3, we can conclude that the final scale of EVD outbreaks in Liberia will get larger when the *β*_1_ (or *β*_2_) increases which is consistent with the actual situation. If effective control measures are not taken, the epidemic will become more serious and there will be much more new infected cases in the future.

### Control measures of EVD in Liberia

Now we consider the effects of existing control measures on EVD in Liberia. There are mainly two kinds of prevention strategies: isolation of the infected individuals (*I*_*S*_, *I*_*P*_, *H*) and safe burial of cases dead but not yet buried (*F*). For comparison, we need to examine the efficiency of isolation of *I*_*S*_, *I*_*P*_, *H* and safe burial of *F*. Firstly, we consider the model with isolation and safe burial measures as follows:
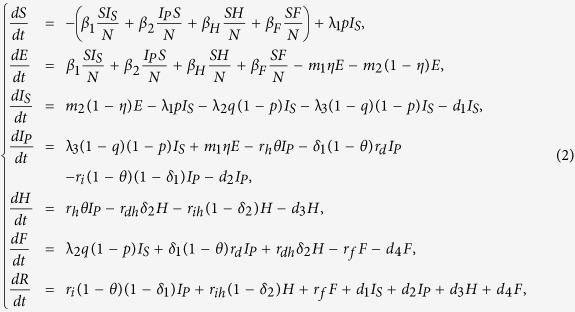
where *d*_1_, *d*_2_, *d*_3_ are the isolation rates of suspected cases, probable cases and hospitalized cases in the community, and *d*_4_ is the safe burial rate of cases dead but not yet buried in funerals. Assume that *d*_1_, *d*_2_, *d*_3_ and *d*_4_ are all non-negative and the remaining parameters are shown in Table 1.

In the following part, we consider the sensitivity analysis of isolation and safe burial on *R*_*c*_ and its expression has the following form:
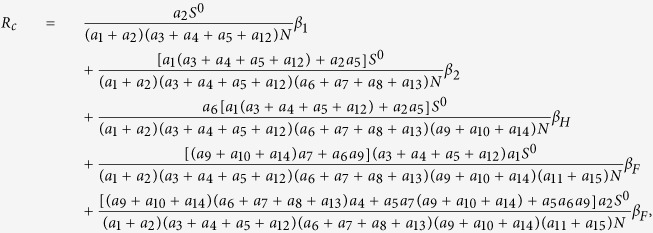
where 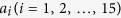 can be found in the **Method**.

**Figure 4:**
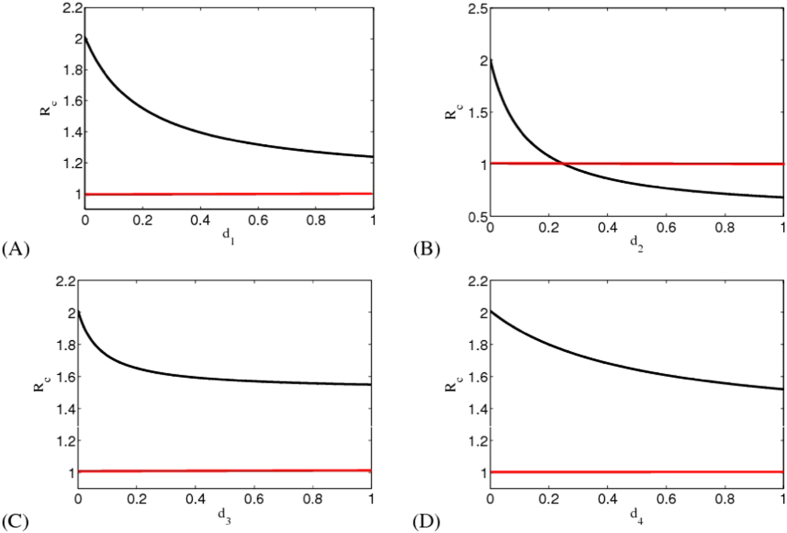
Influence of single control measure on EVD spreading in Liberia. This figure indicates that only take single control measure is not effective for EVD control in Liberia.

We show *R*_*c*_ with respect to *d*_1_ with *d*_2_ = 0, *d*_3_ = 0, *d*_4_ = 0 in Fig. 4(A). Although *d*_1_ = 1, we have that *R*_*c*_ = 1.2344 > 1, which means that only taking control measures on suspected cases (*I*_*S*_) is not enough to control EVD. In the case with *d*_1_ = 0, *d*_3_ = 0, *d*_4_ = 0, we find that *R*_*c*_ may be less than one (see Fig. 4(B)). That is to say when isolation measure on probable cases is sufficient to take, EVD in Liberia will ultimately disappear.

In Fig. 4(C), we check the effect of isolation measure in the hospital on EVD spreading. It can be seen from this figure that *R*_*c*_ = 1.5685 > 1 even if *d*_3_ = 1, which means that only taking control measure in hospital is not enough to eliminate the EVD in Liberia. We also show the influence of safe burial measure on eradication of disease in Fig. 4(D). One concludes that only taking safe burial measure in funerals cannot induce the disappearance of disease due to that *R*_*c*_ = 1.521 > 1 with *d*_4_ = 1.

**Figure 5:**
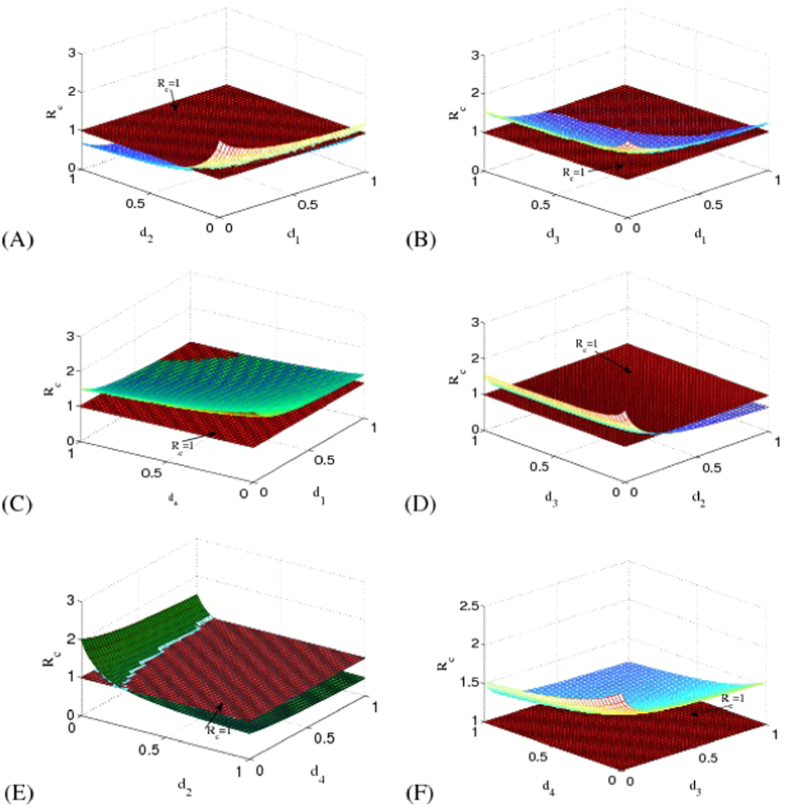
Influences of combined control measures on EVD spreading in Liberia. As seen from this figure, if we take several control measures together, EVD in Liberia may be well controlled.

From the above analysis, we find that taking isolation measure on probable cases may be an effective method to control the prevalence of EVD in Liberia. However, the actual situation in Liberia is that it is nearly impossible to isolate all the probable cases. In that case, it is necessary to combine different control measures together. In Fig. 5, we show the influences of combined control measures on *R*_*c*_. We can see that it is possible to cause basic reproduction number *R*_*c*_ to be less than one for a certain range in parameters space except for Fig. 5(F). Compared with single control measure, combined control strategies are more useful for EVD control in Liberia.

## Discussion

EVD is a lethal disease with a high mortality rate which has caused many deaths. Although the Liberian government has taken some control measures, the number of infectives of EVD increases continuously. Consequently, we consider the contribution of different settings for transmission of EVD in the estimation of *R*_0_ with no effective control measures. Based on the parameters estimation and literature^11^, we obtain *R*_0_ = 2.012, where the term of *R*_0_ concerning the transmission during funeral is about *R*_0*F*_ = 0.736 and the contact transmission with suspected cases (*I*_*S*_) in the community is about 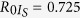 which implies that these two transmission routes play more important roles in EVD transmission in Liberia.

Our model for EVD transmission in Liberia is based on ordinary differential equations (ODEs) which can be analyzed by mathematical analysis and make prediction on the trend of EVD, and thus it has essential differences from the research on EVD by agent-based model or branching process model^25^,^26^. Moreover, different from the previous work^25^,^26^,^27^,^28^,^29^, we divide the infected individuals into two classes: suspected case (*I*_*S*_) and probable case (*I*_*P*_), which is more in line with the actual situations of EVD in Liberia. We estimate basic reproduction number *R*_0_ = 2.012 of EVD, which confirms the results obtained by Castillo-Chavez *et al.* that the basic reproduction number of Ebola in Liberia is in the range of [1.9, 2.4]^30^.

In our results, we estimate that EVD in Liberia may outbreak after 370 days since the time when the first case was confirmed. Moreover, the final size of suspected infectives may achieve 22, 000 cases. That is to say, EVD in Liberia is not well controlled in the current situation. In this case, we need to find effective methods to curb the spread of EVD. Based on sensitivity analysis, we demonstrate that only taking single measure can not control the spread of EVD well, which is consistent with the conclusions posed by Khan *et al.*^27^. Furthermore, we find that taking several control strategies together may be effective for EVD control in Liberia, which highly matches the findings by Merler *et al.* that decrease of incidence at country and county level is attributable to the increasing availability of EVD treatment^25^.

The prevalence of the disease is not optimistic currently and the natural reservoir is still not identified^2^,^6^,^7^,^8^, and thus EVD may outbreak somewhere outside of Africa in the future. In the further study, we will try to define nature reservoir by using mathematical models^8^,^31^. At the same time, the good news is that there are some therapies for EVD^32^, which needs to be well checked on the effectiveness of EVD control. What is more, contact tracing is an effective method in controlling EVD^33^,^34^, and we will do some efforts to examine the influences of human behaviors in EVD control in details.

## Method

### Data

Time series of reported cases were collected from the World Health Organization and the Ministry of Health of Liberia. The data contains the *I*_*S*_, *I*_*P*_ (The definition can be founded in^35^), the deaths and the confirmed cases. Though the data does not contain the patient level information, they provide the best available data on the outbreak of EVD in Liberia. More details on data is available in ref. 24.

### Mathematical model

In order to make our model more reasonable, we must do some assumptions (transmission rules can be seen in Table 3):

- Nearly all the population in Liberia was considered initially as the susceptible;
- Assume no effective prevention measures before November, 7th, 2014;
- If a suspected case goes to see a doctor the suspected case will be considered as a probable case;
- Some misdiagnosed cases will return to be susceptible;
- We only consider the spread in human beings.

**Figure 6:**
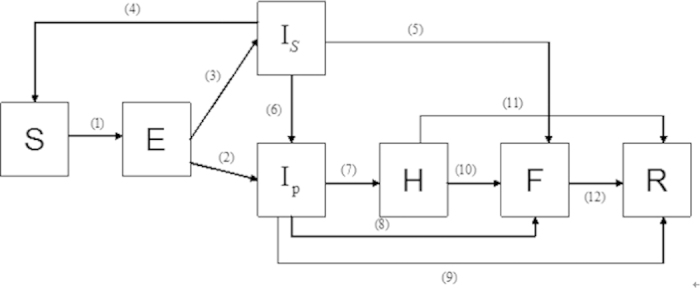
Flow diagram of the compartmental model of EVD transmission in Liberia.

As a result, we arrive at the following equations to model the transmission dynamics of EVD in Liberia without effective control measures (Transmission diagram can be seen from Fig. 6):
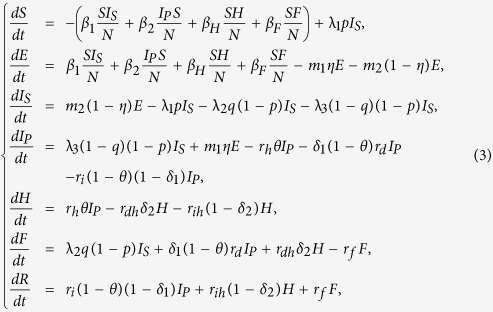
where *S* is number of susceptible individuals; *E*, number of exposed individuals; *I*_*S*_, number of the suspected individuals in the community; *I*_*P*_, number of probable individuals in the community; *H*, number of the hospitalized cases; *F*, number of cases who are dead but not yet buried; *R*, number of individuals removed from the chain of transmission^10^.

Parameter *β*_1_ is transmission coefficient with the suspected cases in the community; *β*_2_, transmission coefficient with the probable cases in the community; *β*_*H*_, transmission coefficient with the hospitalized cases; *β*_*F*_, transmission coefficient during the funerals; *θ*, proportion of suspected cases hospitalized; *p*, misdiagnosed proportion in the suspected cases; *q*, the proportion of suspected cases except the misdiagnosed; *η*, the proportion of exposed cases who enter the *I*_*P*_ compartment; Case-fatality ratio from probable cases to death is *δ*_1_; Case-fatality ratio from hospitalized to death is *δ*_2_; 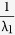, the mean duration of suspected cases return to the susceptible compartment; 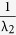, the mean duration of progression from suspected cases to probable cases; 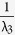, the mean life time of suspected cases; 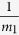, the mean duration of progression from exposed cases to the probable cases; 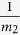, the mean duration of progression from exposed cases to suspected cases; the mean duration of progression from probable cases to hospitalized cases is 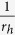; the mean duration of progression from hospitalized to death is 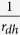; 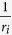 denotes the mean duration of probable cases for survivors; the mean duration from hospitalized to end of infectious for survivors is 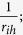 the mean duration from death to burial is 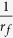; time from probable cases to death is 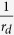.

### The basic reproduction number

Through direct calculation, we obtain that the model (3) has a disease-free equilibrium 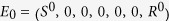, and the formula for *R*_0_ is the spectral radius of the next generation matrix. Following the method described in van den Driessche^23^, we consider the infected compartments satisfied by model (3), which has the following form:
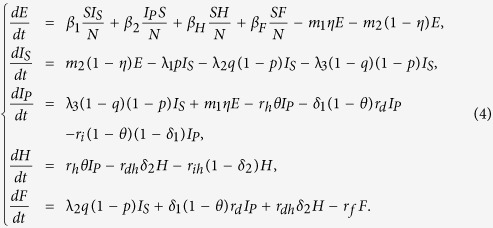


In order to express simply, we do some marks: 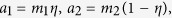

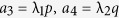

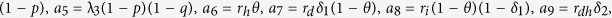

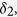

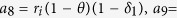

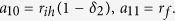


We have the following matrix:
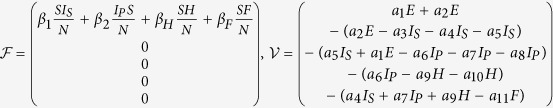
where 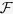 represents the rate of appearance of new infection and 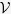 denotes the rate of transfer of individuals. Calculating the derivative of 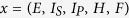, then substituting disease-free equilibrium 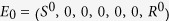 into the variables, we can obtain:
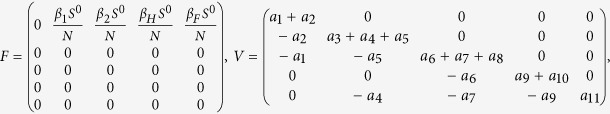


with
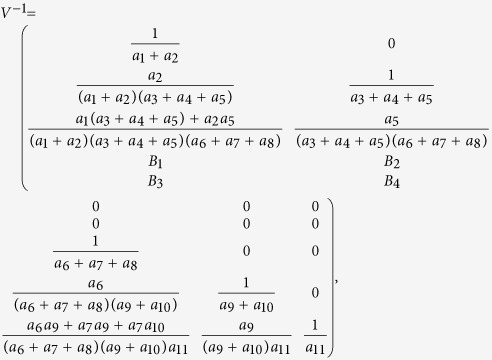
where
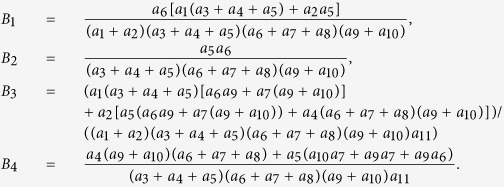


Therefore, the basic reproduction number 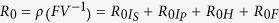, where 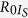, 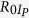, *R*_0*H*_, *R*_0*F*_ are partial reproduction numbers induced by the suspected cases, probable cases, hospitalized cases and dead cases but not yet buried, respectively.

Next we calculate the expressing of the reproduction number (*R*_*c*_) of model (2). For simplicity, we do some marks: *a*_12_ = *d*_1_, *a*_13_ = *d*_2_, *a*_14_ = *d*_3_, *a*_15_ = *d*_4_.

We have the following matrix:
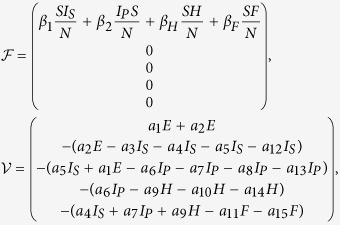
where 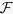 represents the rate of appearance of new infection and 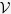 denotes the rate of transfer of individuals. Furthermore, we can obtain:
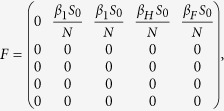

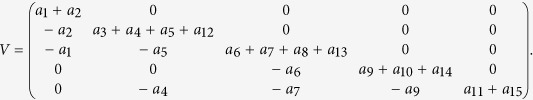


Inverse matrix *V*^−1^ of matrix *V*:
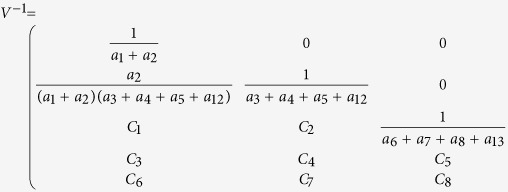

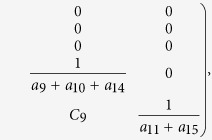
where:
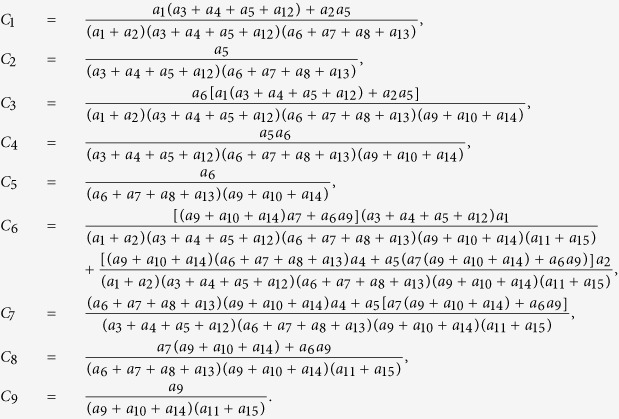

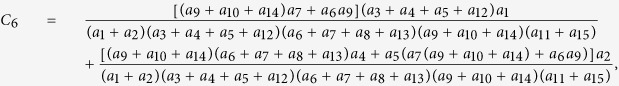


Finally, the basic reproduction number 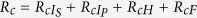, where 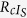, 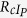, *R*_*cH*_, *R*_*cF*_ are partial reproduction number induced by the suspected cases in the community, probable cases in the community, hospitalized cases and cases dead but not yet buried, respectively.

## Additional Information

**How to cite this article**: Xia, Z.-Q. *et al.* Modeling the transmission dynamics of Ebola virus disease in Liberia. *Sci. Rep.* **5**, 13857; doi: 10.1038/srep13857 (2015).

## Figures and Tables

**Table 1:** Description of parameters in the transmission model (3).

**Parameters description**	**Values**	**Reference**
Size of the Liberia population (*N*)	3441790	36
Proportion of cases hospitalized (*θ*)	0.197	11
Misdiagnosed proportion in the suspected cases (*p*)	0.1	[estimated]
Proportion of suspected cases except the misdiagnosed (*q*)	0.8537	[calculation]
Proportion of exposed cases enter the *I*_*p*_ compartment (*η*)	0.5189	[calculation]
Time of suspected cases return to the susceptible compartmental 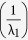	21days	11
Time of suspected cases turn into the probable cases 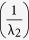	1.5days	[calculation]
The mean life time of suspected cases 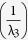	6.68days	[calculation]
Time of exposed cases turn into the probable cases 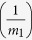	12.00days	11
Time of exposed cases turn into the suspected cases 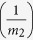	12.00days	11
Time from probable cases enter the hospital 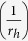	3.24days	11
The mean duration from hospitalized to death 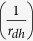	10.07days	11
The mean duration of probable cases for survivors 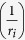	15.00days	[estimated]
Time from hospitalized to end of infectious for survivors 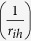	15.88days	11
The mean duration from death to burial 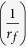	2.01days	11
Time from infection to death 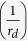	13.31days	11
Case-fatality ratio from infectious to death (*δ*_1_)	0.8	11
Case-fatality ratio from hospitalized to death (*δ*_2_)	0.4	10,11
Transmission coefficient with the suspected in the community (*β*_1_)	0.1102	[estimated]
Transmission coefficient with the probable cases in the community (*β*_2_)	0.12	[estimated]
Transmission coefficient at the hospital (*β*_*H*_)	0.062	11
Transmission coefficient during the funerals (*β*_*F*_)	0.489	11
The initial number of susceptible sheep (*S*′)	3441700	11
The initial number of exposed individuals (*E*′)	20	24
The initial number of suspected individuals 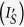	29	24
The initial number of probable individuals 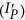	18	24
The initial number of hospitalized individuals (*H*′)	20	24
The initial number of cases dead but not yet buried (*F*′)	11	24
The initial number of *R* (*R*′)	23	24

**Table 2:** Partial rank correlation coefficient (PRCC) for the basic reproduction number *R*_0_ and each input parameter variable.

**Input**	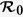	
**parameter**	**PRCC**	***p*** **value**
*β*_1_	0.7843	0.00
*β*_2_	0.9459	0.00
*β*_*h*_	0.3540	0.00
*β*_*f*_	0.0058	0.00
*θ*	−0.0056	0.00
*η*	0.2277	0.00

**Table 3:** Transmission rules of compartmental model (3).

**Number**	**Transition**	**Transition rate**
(1)	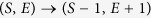	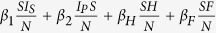
(2)	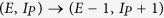	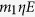
(3)	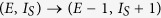	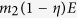
(4)	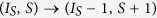	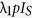
(5)	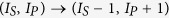	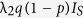
(6)	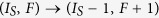	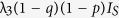
(7)	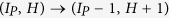	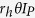
(8)	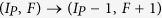	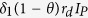
(9)	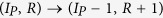	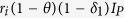
(10)	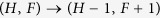	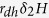
(11)	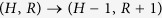	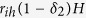
(12)	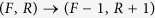	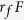
